# Emergent Complex Network Geometry

**DOI:** 10.1038/srep10073

**Published:** 2015-05-18

**Authors:** Zhihao Wu, Giulia Menichetti, Christoph Rahmede, Ginestra Bianconi

**Affiliations:** 1 Beijing Key Lab of Traffic Data Analysis and Mining, School of Computer and Information Technology, Beijing Jiaotong University, Beijing 100044, China; 2 Department of Physics and Astronomy and INFN Sez. Bologna, Bologna University, Viale B. Pichat 6/2 40127 Bologna, Italy; 3 Karlsruhe Institute of Technology, Institute for Theoretical Physics, 76128 Karlsruhe, Germany; 4 School of Mathematical Sciences, Queen Mary University of London, E1 4NS London, United Kingdom

## Abstract

Networks are mathematical structures that are universally used to describe a large variety of complex systems such as the brain or the Internet. Characterizing the geometrical properties of these networks has become increasingly relevant for routing problems, inference and data mining. In real growing networks, topological, structural and geometrical properties emerge spontaneously from their dynamical rules. Nevertheless we still miss a model in which networks develop an emergent complex geometry. Here we show that a single two parameter network model, the growing geometrical network, can generate complex network geometries with non-trivial distribution of curvatures, combining exponential growth and small-world properties with finite spectral dimensionality. In one limit, the non-equilibrium dynamical rules of these networks can generate scale-free networks with clustering and communities, in another limit planar random geometries with non-trivial modularity. Finally we find that these properties of the geometrical growing networks are present in a large set of real networks describing biological, social and technological systems.

Recently, in the network science community^1^,^2^,^3^,^4^, the interest in the geometrical characterizations of real network datasets has been growing. This problem has indeed many applications related to routing problems in the Internet^5^,^6^,^7^,^8^, data mining and community detection^9^,^10^,^11^,^12^,^13^,^14^. At the same time, different definitions of network curvatures have been proposed by mathematicians^15^,^16^,^17^,^18^,^19^,^20^,^21^,^22^,^23^,^24^, and the characterization of the hyperbolicity of real network datasets has been gaining momentum thanks to the formulation of network models embedded in hyperbolic planes^25^,^26^,^27^,^28^,^29^, and by the definition of delta hyperbolicity of networks by Gromov^22^,^30^^–^32. This debate on geometry of networks includes also the discussion of useful metrics for spatial networks^33^,^34^ embedded into a physical space and its technological application including wireless networks^35^.

In the apparently unrelated field of quantum gravity, pregeometric models, where space is an emergent property of a network or of a simplicial complex, have attracted large interest over the years^36^,^37^,^38^,^39^,^40^,^41^,^42^,^43^. Whereas in the case of quantum gravity the aim is to obtain a continuous spacetime structure at large scales, the underlying simplicial structure from which geometry should emerge bears similarities to networks. Therefore we think that similar models taylored more specifically to our desired network structure (especially growing networks) could develop emergent geometrical properties as well.

Here our aim is to propose a pregeometric model for emergent complex network geometry, in which the non-equilibrium dynamical rules do not take into account any embedding space, but during its evolution the network develops a certain heterogeneous distribution of curvatures, a small-world topology characterized by high clustering and small average distance, a modular structure and a finite spectral dimension.

In the last decades the most popular framework for describing the evolution of complex systems has been the one of growing network models^1^,^2^,^3^. In particular growing complex networks evolving by the preferential attachment mechanism have been widely used to explain the emergence of the scale-free degree distributions which are ubiquitous in complex networks. In this scenario, the network grows by the addition of new nodes and these nodes are more likely to link to nodes already connected to many other nodes according to the preferential attachment rule. In this case the probability that a node acquires a new link is proportional to the degree of the node. The simplest version of these models, the Barabasi-Albert (BA) model^44^, can be modified^1^,^2^,^3^ in order to describe complex networks that also have a large clustering coefficient, another important and ubiquitous property of complex networks that characterizes small-world networks^45^ together with the small typical distance between the nodes. Moreover, it has been recently observed^46^,^47^ that growing network models inspired by the BA model and enforcing a high clustering coefficient, using the so called triadic closure mechanism, are able to display a non trivial community structure^48^,^49^. Finally, complex social, biological and technological networks not only have high clustering but also have a structure which suggests that the networks have an hidden embedding space, describing the similarity between the nodes. For example the local structure of protein-protein interaction networks, analysed with the tools of graphlets, suggests that these networks have an underlying non-trivial geometry^50^,^51^.

Another interesting approach to complex networks suggests that network models evolving in a hyperbolic plane might model and approximate a large variety of complex networks^28^,^29^. In this framework nodes are embedded in a hidden metric structure of constant negative curvature that determine their evolution in such a way that nodes closer in space are more likely to be connected.

But is it really always the case that the hidden embedding space is causing the network dynamics or might it be that this effective hidden metric space is the outcome of the network evolution?

Here we want to adopt a growing network framework in order to describe the emergence of geometry in evolving networks. We start from non-equilibrium growing dynamics independent of any hidden embedding space, and we show that spatial properties of the network emerge spontaneously. These networks are the skeleton of growing simplicial complexes that are constructed by gluing together simplices of given dimension. In particular in this work we focus on simplicial complexes built by gluing together triangles and imposing that the number of triangles incident to a link cannot be larger than a fixed number 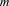 that parametrizes the network dynamics. In this way we provide evidence that the proposed stylized model, including only two parameters, can give rise to a wide variety of network geometries and can be considered a starting point for characterizing emergent space in complex networks. Finally we compare the properties of real complex system datasets with the structural and geometric properties of the growing geometrical model showing that despite the fact that the proposed model is extremely stylized, it captures main features observed in a large variety of datasets.

## Results

Metric spaces satisfy the triangular inequality. Therefore in spatial networks we must have that if a node 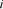 connects two nodes (the node 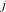 and the node 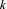), these two must be connected by a path of short distance. Therefore, if we want to describe the spontaneous emergence of a discrete geometric space, in absence of an embedding space and a metric, it is plausible that starting from growing simplicial complexes should be an advantage. These structures are formed by gluing together complexes of dimension 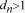, i.e. fully connected networks, or cliques, formed by 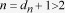 nodes, such as triangles, tetrahedra etc. For simplicity, let us here consider growing networks constructed by addition of connected complexes of dimension 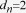, i.e. triangles. We distinguish between two cases: the case in which a link can belong to an arbitrarily large number of triangles (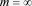), and the case in which each link can belong at most to a finite number 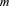 of triangles. In the case in which 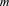 is finite we call the links to which we can still add at least one triangle unsaturated. All the other links we call saturated.

**Figure 1:**
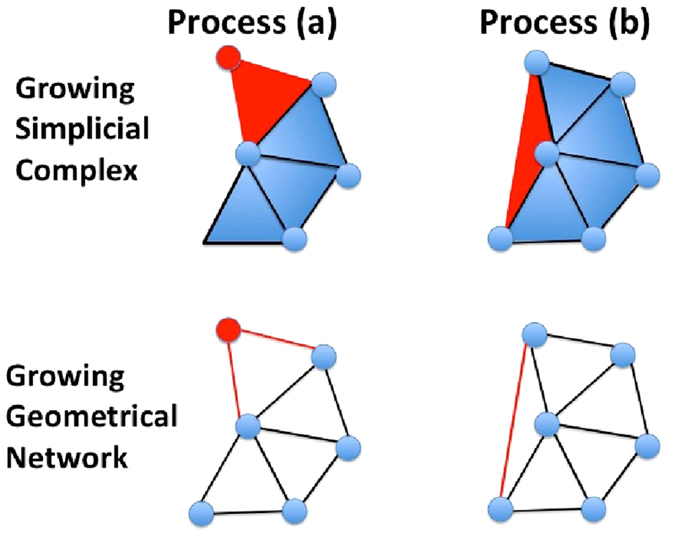
The two dynamical rules for constructing the growing simplicial complex and the corresponding growing geometrical network . In process (**a**) a single triangle with one new node and two new links is added to a random unsaturated link, where by unsaturated link we indicate a link having less than 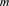 triangles incident to it. In process (**b**) with probability 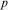 two nodes at distance two in the simplicial complex are connected and all the possible triangles that can link these two nodes are added as long as this is allowed (no link acquires more than 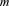 triangles incident to it). The growing geometrical network is just the network formed by the nodes and the links of the growing simplicial complex. In the Figure we show the case in which 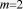.

To be precise, we start from a network formed by a single triangle, a simplex of dimension 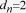. At each time we perform two processes (see Fig. 1).

• *Process (a)-* We add a triangle to an unsaturated link 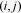 of the network linking node 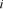 to node 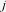. We choose this link randomly with probability 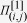 given by


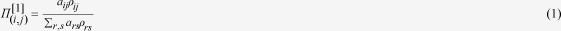


where 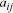 is the element 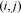 of the adjacency matrix ***a*** of the network, and where the matrix element 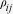 is equal to one (i.e. 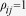) if the number of triangles to which the link 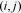 belongs is less than 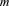, otherwise it is zero (i.e. 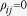). Having chosen the link 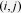 we add a node 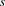, two links 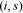 and 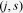 and the new triangle linking node 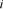, node 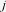 and node 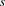.

• *Process (b)-* With probability 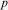 we add a single link between two nodes at hopping distance 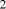, and we add all the triangles that this link closes, without adding more than 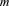 triangles to each link. In order to do this, we choose an unsaturated link 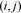 with probability 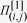 given by Eq. (1), then we choose one random unsaturated link adjacent either to node 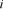 or node 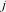 as long as this link is not already part of a triangle including node 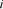 and node 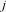. Therefore we choose the link 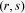 with probability 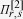 given by


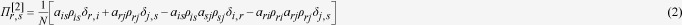


where 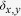 is the Kronecker delta and 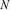 is the normalization constant. Let us assume without loss of generality that the chosen link 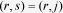. Then we add a link 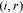 and all the triangles passing through node 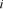 and node 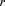 as long as this process is allowed (i.e. if by doing so we do not add more than 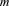 triangles to each link). Otherwise we do nothing.

With the above algorithm (see Supplementary Information for the MATLAB code) we describe a growing simplicial complex formed by adding triangles. From this structure we can extract the corresponding network where we consider only the information about node connectivity (which node is linked to which other node). We call this network model the geometrical growing network. In Fig. 1 we show schematically the dynamical rules for building the growing simplicial complexes and the geometrical growing networks that describe its skeleton.

Let us comment on two fundamental limits of this dynamics. In the case 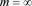, 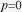, the network is scale-free and in the class of growing networks with preferential attachment. In fact the probability that we add a link to a generic node 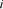 of the network using process 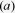 is simply proportional to the number of links connected to it, i.e. its degree 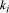. Therefore, the mean-field equations for the degree 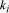 of a generic node 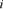 are equal to the equations valid for the BA model, i.e. they yield a scale-free network with power-law exponent 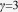. Actually this limit of our model was already discussed in^52^ as a simple and major example of scale-free network. For 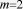, instead, the degree distribution can be shown to be exponential (see Methods and Supplementary material for details). The Euler characteristic 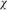 of our simplicial complex and the corresponding network is given by


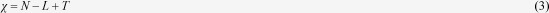


where 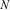 indicates the total number of nodes, 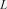 the total number of links and 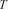 the total number of triangles in the network. For 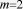 and any value of 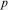, or for 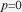 and any value of 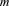 the networks are planar graphs since the non-planar subgraphs 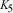 (complete graph of five nodes) and 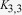 (complete bipartite graph formed by two sets of three nodes) are excluded from the dynamical rules (see Methods for details). Therefore in these cases we have an Euler characteristic 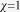 (in fact here we do not count the external face).

**Figure 2:**
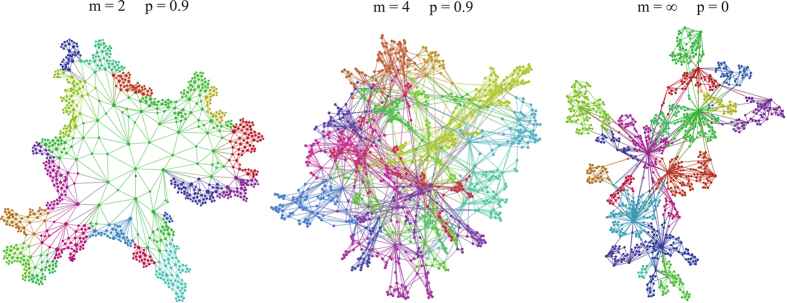
The growing geometrical network model can generate networks with different topology and geometry. In the case 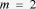, 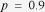 a random planar geometry is formed. In the case 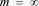, 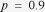 a scale-free network with power-law exponent 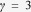 and non trivial community structure and clustering coefficient is formed. In the intermediate case 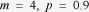 a network with broad degree distribution, small-world properties and finite spectral dimension is formed. The colours here indicate division into communities found by running the Leuven algorithm^53^.

In general the proposed growing geometric network model can generate a large variety of network geometries. In Fig. 2 we show a visualization of single instances of the growing geometrical networks in the cases 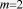, 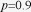 (random planar geometry), 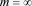, 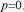 (scale-free geometry), and 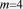, 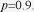


The growing geometrical network model has just two parameters 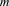 and 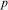. The role of the parameter 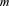 is to fix the maximal number of triangles incident on each link. The role of the parameter 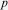 is to allow for a non-trivial K-core structure of the network. In fact, if 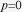 the network can be completely pruned if we remove nodes of degree 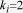 recursively, similarly to what happens in the BA model, while for 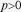 the geometrical growing network has a non-trivial 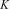-core. Moreover the process 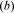 can be used to “freeze” some region of the network. In order to see this, let us consider the role of the process 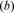 occurring with probability 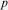 in the case of a network with 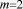. Then for 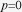, each node will increase its connectivity indefinitely with time having always exactly two unsaturated links attached to it. On the contrary, if 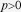 there is a small probability that some nodes will have all adjacent links saturated, and a degree that is frozen and does not grow any more. A typical network of this type is shown for 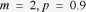 in Fig. 2 where one can clearly distinguish between an active boundary of the network where still many triangles can be linked and a frozen bulk region of the network.

**Figure 3:**
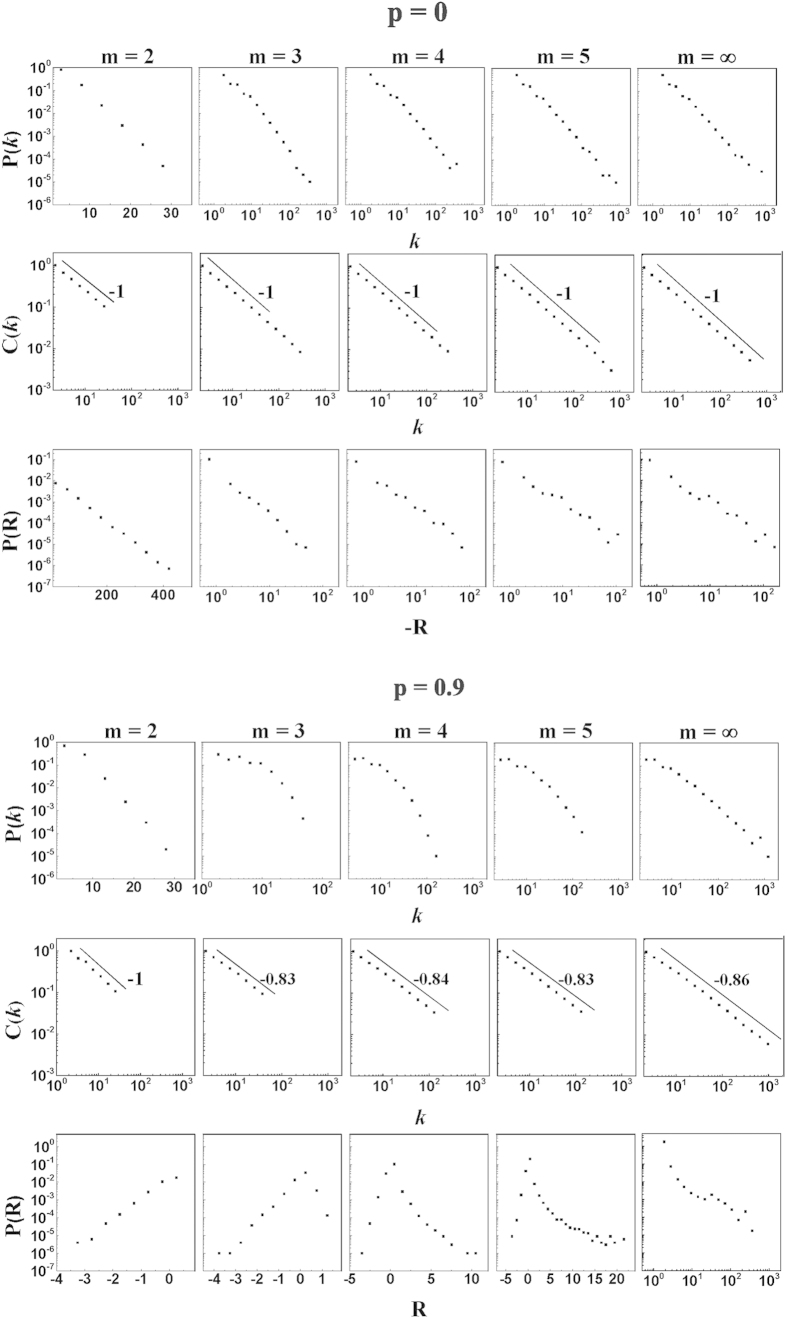
Local properties of the growing geometrical model. We plot the degree distribution 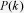, the distribution of curvature 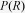, and the average clustering coefficient 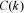 of nodes of degree 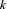 for networks of sizes 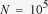, parameter 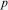 chosen as either 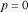 or 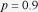, and different values of 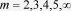. The network has exponential degree distribution for 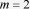 and scale-free degree distribution for 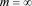. For 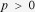 and 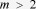 it shows broad degree distribution. The networks are always hierarchical, to the extent that 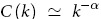 with 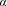 shown in the figure. The distribution of curvature 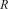 is exponential for 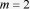 and scale-free for 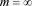. For 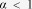 the curvature has a positive tail.

The geometrical growing networks have highly heterogeneous structure reflected in their local properties. For example, the degree distribution is scale-free for 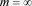 and exponential for 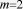 for any value of 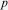. Moreover for finite values of 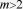 the degree distribution can develop a tail that is broader for increasing values of 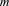 (see Fig. 3). Furthermore, in Fig. 3 we plot the average clustering coefficient 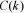 of nodes of degree 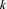 showing that the geometrical growing networks are hierarchical^49^, they have a clustering coefficient 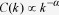 with values of 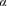 that are typically 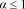.

Another important and geometrical local property is the curvature, defined on each node of the network. For either 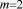 and any value of 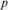 or for 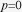 and any value of 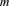, the generated graph is a planar network of which all faces are triangles. Therefore we consider the curvature 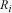
19,20,21,22 given by


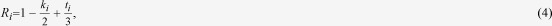


where 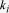 is the degree of node 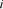, and 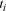 is the number of triangles passing through node 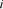.

We observe that the definition of the curvature satisfies the Gauss-Bonnet theorem


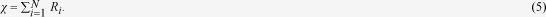


For a planar network, for bulk nodes which have 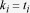 the curvature reduces to


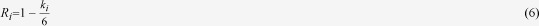


and for nodes at the boundary for which 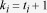, it reduces to


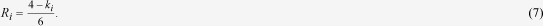


Note that the expression in Eq. (7) is also valid for 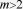 as long as 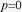. In fact for these networks only process 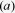 takes place and it is easy to show that 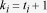. This simple relation between the curvature 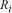 and the degree 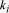 allows to characterize the distribution of curvatures in the network easily. The curvature is intuitively related to the degree of the node. As all triangles are isosceles, a bulk node with degree six has zero curvature. In fact the sum of the angles of the triangles incident to the node is 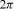. Otherwise the sum is smaller or larger than 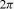 resulting in positive or negative curvature respectively. The argument works similarly for the nodes at the boundary.

For 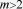 and 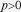 the networks are not planar anymore, and the definition of curvature is debated 15,16,17,18. Here we decided to continue to use the definition given by Eq. (4). This is equivalent to the definition of curvature by Oliver Knill^23^,^24^, in which the curvature 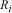 at a node 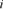 is defined as


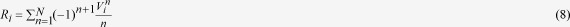


where 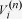 are the number of simplices of 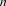 nodes and dimension 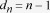 to which node 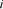 belongs. In fact the definition of curvature given by Eq. (4) is equivalent to the definition given by Eq. (8) if we truncate the sum in Eq. (8) to simplices of dimension 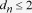, i.e. we consider only nodes, links and triangles since these are the original simplices building our network.

For 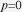 the curvature distribution is dominated by a negative unbounded tail that is exponential in the case 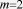 and power-law in the case 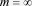. In particular while the average curvature is 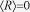 for 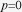 and any value of 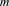, in the limit 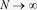 the fluctuations around this average are finite (i.e. 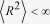) for 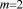, and infinite (i.e. 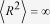) for 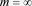. We note here that in the BA model the clustering coefficient 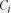 of any node 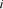 vanishes in the large network limit, therefore the curvature 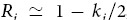, and the curvature distribution has a power-law negative tail and diverging 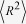 in the large network limit, similarly to the case 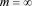 and 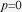 of the present model.

For a general value of 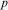, we can assume that the average clustering 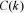 of nodes of degree 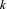, scales as 
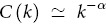
. Then the average number of triangles 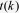 of nodes of degree 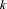, scales as 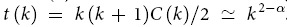. Therefore, for large 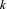 and as long as 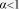 the average curvature of nodes of degree 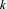 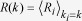, is dominated by the contribution of triangles and scales like 
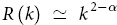
 with a positive tail for large values of 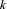. This allows us to distinguish the phase diagram in two different regions according to the value of the exponent 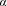: the case 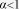 in which the curvature has a positive tail, and the case 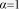 in which the curvature can have a negative tail.

We make here two main observations. First of all, with the definition of the curvature given byEq. (4), our network model has heterogeneous distribution of curvatures. Therefore here we are characterizing highly heterogeneous geometries and the geometrical growing network does not have a constant curvature. This is one of the main differences of the present model compared to network models embedded in the hyperbolic plane^28^,^29^. In particular all the networks with 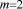 or 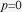 have 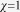 and therefore the average curvature is zero in the thermodynamical limit, but they have a curvature distribution with an unbounded negative tail that can be either exponential for 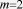 (i.e. 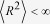) or scale-free as for the case 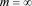 (i.e. 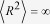).

We illustrate this in Fig. 3 where we plot the distribution 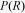 of curvatures for different specific models of growing geometrical networks for 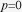 and 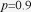 for different values of 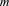. We show that for 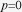 the negative tail can be either exponential or scale-free. For 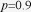 we have for 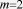 a negative exponential tail and for 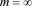 a positive scale-free tail of the curvature distribution consistent with a value of the exponent 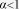 and a power-law degree distribution.

**Figure 4:**
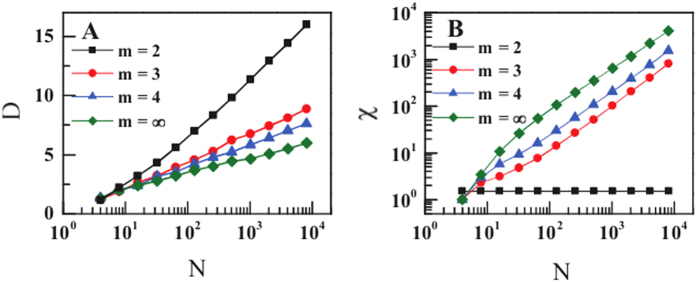
Maximum distance 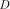 from the initial triangle and Euler characteristic 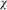 as a function of the network size 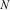
. The geometrical network model is growing exponentially, with 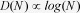. Here we show the data 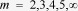 and 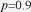 (panel A). The Euler characteristic 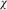 is given by 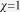 for 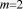 and 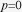 and grows linearly with 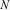 for the other values of the parameters of the model (panel B).

Our second observation is that the case 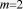 and 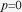 is significantly different from the case 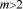 and 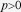. In fact for 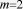 and for 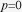 the Euler characteristic of the network is 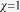 and never increases in time (see Methods for details), while for the case 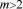, 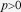 we expect 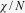 to go to a finite limit as 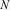 goes to infinity. In Fig. 4 the numerical results of the Euler characteristic 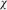 as a function of the network size 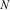 shows that, for 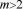 and 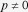, 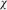 grows linearly with 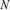. The quantity 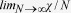 gives the average curvature in the network and is therefore zero for 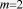 and 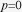.

The generated topologies are small-world. In fact they combine high clustering coefficient with a typical distance between the nodes increasing only logarithmically with the network size. The exponential growth of the network is to be expected by the observation that in these networks we always have that the total number of links as well as the number of unsaturated links scale linearly with time. This corresponds to a physical situation in which the “volume” (total number of links) is proportional to the “surface” (number of unsaturated links). Therefore we should expect that the typical distance of the nodes in the network should grow logarithmically with the network size 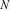. In order to check this, in Fig. 4 we give 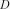, the average distance of the nodes from the initial triangle over the different network realisations as a function of the network size 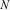. From this figure it is clear that asymptotically in time 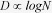, independently of the value of 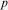 and 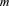.

**Figure 5:**
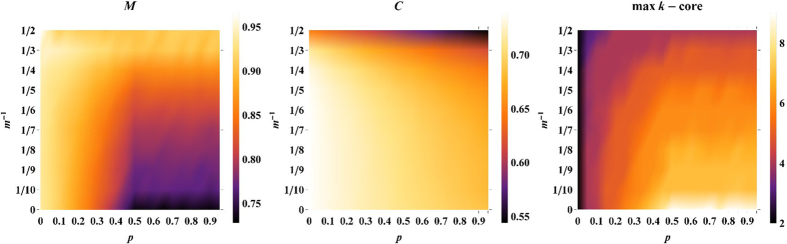
Modularity and clustering of the growing geometrical model. The modularity 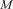 calculated using the Leuven algorithm^53^ on 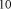 realisations of the growing geometrical network of size 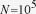 is reported as a function of the parameters 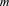 and 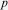 of the model. Similarly the average local clustering coefficient 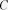 calculated over 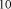 realisations of the growing geometrical networks of size 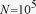 is reported as a function of the parameters 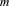 and 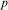. The value of 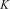 of the maximal 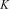-core is shown for a network of 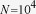 nodes as a function of 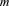 and 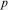. These results show that the growing geometrical networks have finite average clustering coefficient together with non-trivial community and 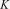-core structure on all the range of parameters 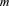 and 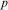.

The effects of randomness and emergent locality in these networks are reflected by their cluster structure, revealed by the lower bound on their maximal modularity measured by running efficient community detection algorithms^53^ (Fig. 5). Moreover also their clustering coefficient provides evidence for their emergent locality (Fig. 5). Finally we observe that for 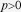 the network develops also a non-trivial K-core structure. In order to show this in Fig. 5 we also plot the value of 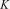 corresponding to the maximal 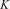-core of the network. As we already mentioned, for 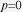 we have 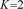 and the network can be completely pruned by removing the triangles recursively. For 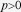 instead, the maximal 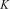-core can have a much larger value of 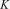, as shown in Fig. 5 for a network of 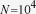 nodes.

Therefore these structures are different from the small world model to the extent that they are always characterised by a non-trivial community and 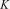-core structure.

**Figure 6:**
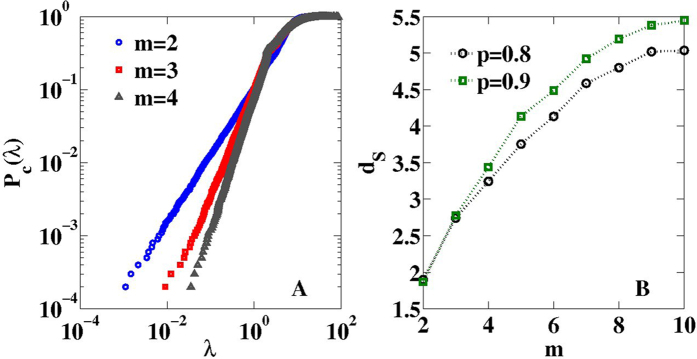
The spectral dimension of the geometrical growing networks. Asymptotically in time, the geometrical growing networks have a finite spectral dimension. Here we show typical plots of the spectral density of networks with 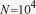 nodes, 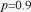 and 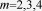 (panel A). In panel B we plot the fitted spectral dimension for 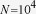 averaged over 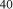 network realizations for 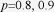.

The geometrical growing network is growing exponentially, so the Hausdorff dimension is infinite. Nevertheless, these networks develop a finite spectral dimension 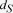 as clearly shown in Fig. 6, for 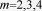 and 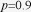. We have checked that also for other values of 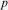 the spectral dimension remains finite. This is a clear indication that these networks have non-trivial diffusion properties.

**Figure 7:**
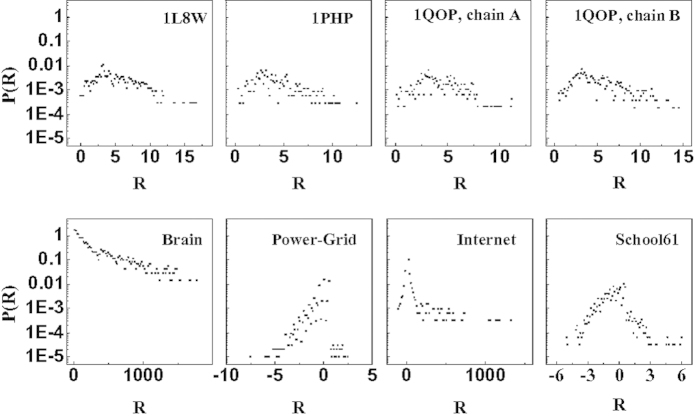
Curvature distribution in real datasets. We plot the distribution 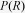 in a a variety of datasets with additional structural and local properties shown in Table 1.

The geometrical growing network model is therefore a very stylized model with interesting limiting behaviour, in which geometrical local and global parameters can emerge spontaneously from the non-equilibrium dynamics. Moreover here we compare the properties of the geometric growing network with the properties of a variety of real datasets. In particular we have considered network datasets coming from biological, social, and technological systems and we have analysed their properties. In Table 1 we show that in several cases large modularity, large clustering, small average distance and non-trivial maximal 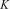-core structure emerge. Moreover, in these datasets a non-trivial distribution of curvature (defined as in Eq. (4)) is present, showing either negative or positive tail (see Fig. 7). Finally the Laplacian spectrum of these networks also displays a power-law tail from which an effective finite spectral dimension can be calculated (see Table 1 and Supplementary Information for details). This shows that the geometrical growing network models have many properties in common with real datasets, describing biological, social, and technological systems, and should therefore be used and modified to model several real network datasets.

## Discussion

In conclusion, this paper shows that growing simplicial complexes and the corresponding growing geometrical networks are characterized by the spontaneous emergence of locality and spatial properties. In fact small-world properties, non-trivial community structure, and even finite spectral dimensions are emerging in these networks despite the fact that their dynamical rules do not depend on any embedding space. These growing networks are determined by non-equilibrium stochastic dynamics and provide evidence that it is possible to generate random complex self-organized geometries by simple stochastic rules.

An open question in this context is to determine the underlying metric for these networks. In particular we believe that the investigation of the hyperbolic character of the models with 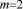 and 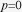 (that have zero average curvature but a negative third moment of the distribution of curvature) should be extremely interesting to shed new light on “random geometries” in which the curvature can have finite or infinite deviations from its average. A full description of their structure using tools of geometric group theory could be envisaged to solve this problem. This analysis could be facilitated also by the study of the dual network in which each triangle is a node of maximal degree 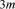. In fact each edge of the triangle is at most incident to other 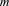 triangles in the geometrical growing network.

Furthermore we mention that the model can be generalized in two main directions. On the one hand the model can be extended by considering geometrical growing networks built by gluing together simplices of higher dimension. On the other hand, one can explore methods to generate networks that have a finite Hausdorff dimension, i.e. that they have a typical distance between the nodes scaling like a power of the total number of nodes in the network. Another interesting direction of further theoretical investigation is to consider the equilibrium models of networks (ensembles of networks) in which a constraint on the total number of triangles incident to a link is imposed, similarly to recent works that have considered ensembles with given degree correlations and average clustering coefficient 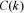 of nodes of degree 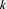
54.

Finally the geometrical growing network is a very stylized model and includes the essential ingredients for describing the emergence of locality of the interactions in complex networks and can be used in a variety of fields in which networks and discrete spaces are important, including complex networks with clustering such as biological, social, and technological networks.

## Methods

### Degree distribution of 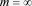 and 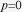 -

In the case 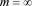 and 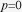 the geometrical growing network model is reduced to the model proposed in^52^. Here we show the derivation of the scale-free distribution in this case for completeness. In the geometrical growing network with 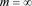 and 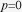 at each time a random link is chosen and a new node attaches two links to the two ends of it. Therefore the probability that at time 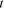 a new link is attached to a given node of degree 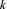 is given by 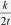. Using this result we can easily write the master equation for the number of nodes 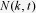 of degree 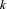 at time 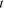,


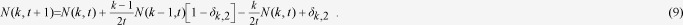


Since the network is growing, asymptotically in time the number of nodes of degree 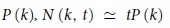 will be proportional to the degree distribution 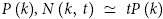, 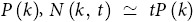, where the total number of nodes in the network is 
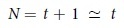
. Therefore, substituting this scaling in Eq. (9) we get


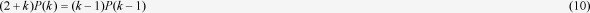


for every 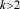, while 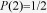 yielding the solution


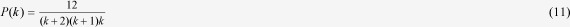


for 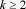, which is equal to the degree distribution of the BA model with minimal degree equal to 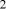, i.e. scale-free with power-law exponent 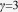. Here we observe that the curvature of the nodes is in this case 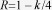, therefore 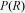 has a power-law negative tail, i.e. 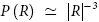 for 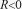 and 
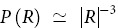
. Moreover we have 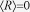 (consistent with 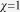) but 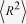 is diverging with the network size 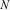.

### Degree distribution of 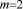 for 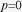 -

The degree distribution for 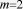 is exponential for any value of 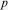. Here we discuss the simple case 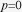 leaving the treatment of the case 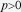 to the Supplementary Information. For 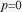 every node has exactly two unsaturated links. The total number of unsaturated links is 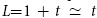 at large time 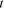. Therefore the average number of links that a node gains at time 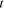 by process 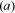 is given by 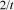 for 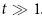. The master equations for the average number of nodes 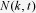 that have degree 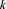 at time 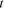 are given by


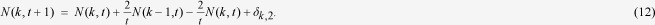


In the large time limit, in which 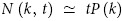, the degree distribution 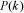 is given by


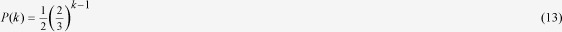


for 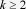. The curvature 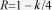 is therefore in average 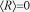 in the limit 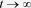 with finite second moment 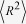.

### Euler characteristic 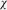 of geometrical growing network with either 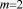 or 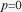 -

The Euler characteristic of the geometrical growing networks with 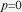 is 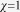 at every time. In fact we start from a single triangle, therefore at 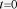 we have 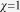. At each time step we attach a new triangle to a given unsaturated link, therefore we add one new node, two new links, and one new triangle, so that 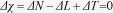. Hence 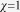 for every network size. For 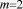 also the process 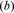 does not increase the Euler characteristic. In fact in this case when the process 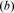 occurs, and 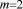, we add only one new link and one new triangle, therefore 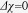 also for this process. Instead in the case 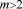 and 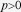, process 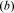 always adds a single link but the number of triangles that close is in average greater than one, therefore the Euler characteristic 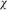 grows linearly with the network size 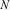.

### Definition of Modularity 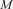 -

The modularity 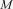 is a measure to evaluate the significance of the community structure of a network. It is defined^48^ as


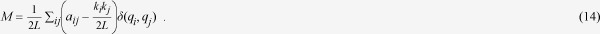


Here, 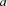 denotes the adjacency matrix of the network, 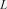 the total number of links, and 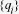, where 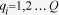, indicates to which community the node 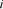 belongs. Finding the network partition that optimizes modularity is a NP hard problem. Therefore different greedy algorithms have been proposed to find the community structure such as the Leuven method^53^ that we have used in this study. The modularity found in this way is a lower bound on the maximal modularity of the network.

### Definition of the Clustering coefficient-

The clustering coefficient is given by the probability that two nodes, both connected to a common node, are also connected. In the context of social networks, it describes the probability that a friend of a friend is also your friend. The local clustering coefficient 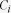 of node 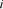 has been defined as the probability that two neighbours of the node 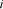 are neighbours of each other,


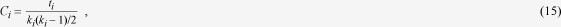


where 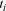 is the number of triangles passing through node 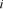, and 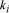 is the degree of node 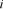.

### Definition of the 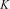 -core-

We define the 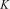-core of a network as the maximal subgraph formed by the set of nodes that have at least 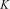 links connecting them to the other nodes of the 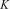-core. The 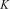-core of a network can be easily obtained by pruning a given network, i.e. by removing iteratively all the nodes 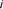 with degree 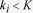.

### Definition of the spectral dimension of a network-

The Laplacian matrix of the network 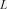 has elements


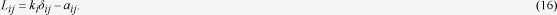


If the density of eigenvalues 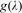 of the Laplacian scales like


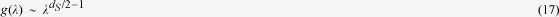


with 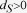, for small values of 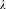, then 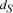 is called the spectral dimension of the network. For regular lattices in dimension 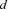 we have 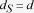. Clearly, if the spectral dimension of a network is well defined, then the cumulative distribution 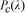 scales like


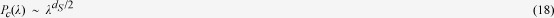


for small values of 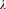.

### Real datasets

We analysed a large variety of biological, technological and social datasets. In particular we have considered the brain network of co-activation^55^^55^, 4 protein contact maps^58^ (see Supplementary Information for details on the data analysis), the Internet at the Autonomous System level^56^, the US power-grid^45^, and a social network of friendship between high-school students coming from the Add Health dataset AddHealth.

## Author Contributions

C. R. and G.B. designed the research, Z. W., G. M. and G. B. wrote the codes, Z. W. and G. M. prepared figures, C. R. and G. B. wrote the main manuscript text, all authors reviewed the manuscript.

## Additional Information

**How to cite this article**: Wu, Z. *et al.* Emergent Complex Network geometry. *Sci. Rep.* **5**, 10073; doi: 10.1038/srep10073 (2015).

## Figures and Tables

**Table 1:** Table showing the structural properties of a variety of real datasets.

Datasets	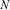	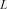	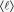	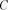	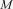	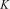	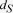
1L8W (protein)	294	1608	5.09	0.52	0.643	7	1.95
1PHP (protein)	219	1095	4.31	0.54	0.638	6	2.02
1AOP chain A (protein)	265	1363	4.31	0.53	0.644	7	2.01
1AOP chain B (protein)	390	2100	4.94	0.54	0.685	7	2.03
Brain-(coactivation) 55	638	18625	2.21	0.384	0.426	46	4.25
Internet 57	22963	48436	3.8	0.35	0.652	25	5.083
Power-grid 45	4941	6594	19	0.11	0.933	5	2.01
Add Health (school61) 58	1743	4419	6	0.22	0.741	6	2.97

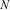
 indicates the total number of nodes, 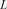 the total number of links, 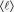 the average shortest distance between the nodes, 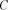 the average local clustering coefficient, 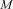 the modularity found by the Leuven algorithm^53^, 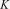 the maximal 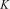-core, and 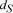 the spectral dimension of the networks. The average shortest distance 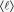 can be checked to be of the same order of magnitude as 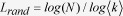 which is the average shortest distance in a random network with the same density of links as the real dataset. The average local clustering coefficient 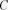 can be checked to be much larger than 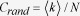 indicating the average clustering coefficient of a random network with the same density of links as the real dataset. For the implications of the finite spectral dimension of proteins on their stability see^59^. The references indicate the source of the data (for the four contact maps of the considered proteins, extracted from^58^ see Supplementary Information for details).
